# Supplementary material for: Widespread impact-generated porosity in early planetary crusts
Source: Nat Commun. 2022 Aug 16;13:4817. doi: 10.1038/s41467-022-32445-3 (PMC9381781; doi:10.1038/s41467-022-32445-3)
Supplement: Supplementary file 1 — Supplementary Information [file 41467_2022_32445_MOESM1_ESM.pdf]

*Nature Communications*

Supplementary Information for

# Widespread Impact-Generated Porosity in Early Planetary Crusts

Sean E. Wiggins[1]*, Brandon C. Johnson[1,2], Gareth S. Collins[3], H. Jay Melosh[1,2,†], Simone Marchi[4]

[1]Department of Earth, Atmospheric, and Planetary Sciences, Purdue University, West Lafayette, IN 47907, USA.

[2]Department of Physics and Astronomy, Purdue University, West Lafayette, IN 47907, USA

[3]Department of Earth Science and Engineering, Imperial College London, SW7 2AZ, United Kingdom.

[4]Southwest Research Institute, Boulder, Colorado 80302 USA.

†Deceased 9/11/2020

## Contents of this file

Supplementary Text 1 to 4
Supplementary Figure 1 to 4
Supplementary Tables 1 to 2

## Introduction

The main purposes of the supplemental text here is to provide detailed simulation input values vital for reproducing our results, and to report our results from simulations examining tensile porosity generation and elimination in a pre-porous crust. Our simulation values are given below in table and written formats. The results from our pre-porous crust simulation are given below as Supplementary Figure 2 with accompanying text that provides some helpful context. However, we have additionally included some small discussion on varying thermal gradients and further visual confirmation on the difference our new routines have on modeling porosity generation from hypervelocity impacts.

### Supplementary Text 1. Simulation inputs:

We model vertical impacts of spherical basaltic impactors on flat, nonporous, basaltic targets. We use an equation of state (EOS) table for basalt generated with the M-ANEOS (Analytic Equations of State) equation of state package[44], and a rock-like strength[33] and damage model[33] for both the impactor and the target. The parameters used to generate the EOS table were taken from [36], modified to fix a typo for the energy of vaporization (1.1x$10^{11}$ erg/g) as well as to raise the solid-solid phase transition pressure to 20 GPa from 5 GPa, in order to eliminate spurious behavior around the transition pressure. A comparison of the Hugoniot curves for the revised basalt EOS and that of Pierazzo et al. (2005) is shown in Supplementary Figure 1. The

revised basalt EOS is a better fit to experimental shock wave data[45,46] than the previous version for the density and pressure range of relevance to this study.

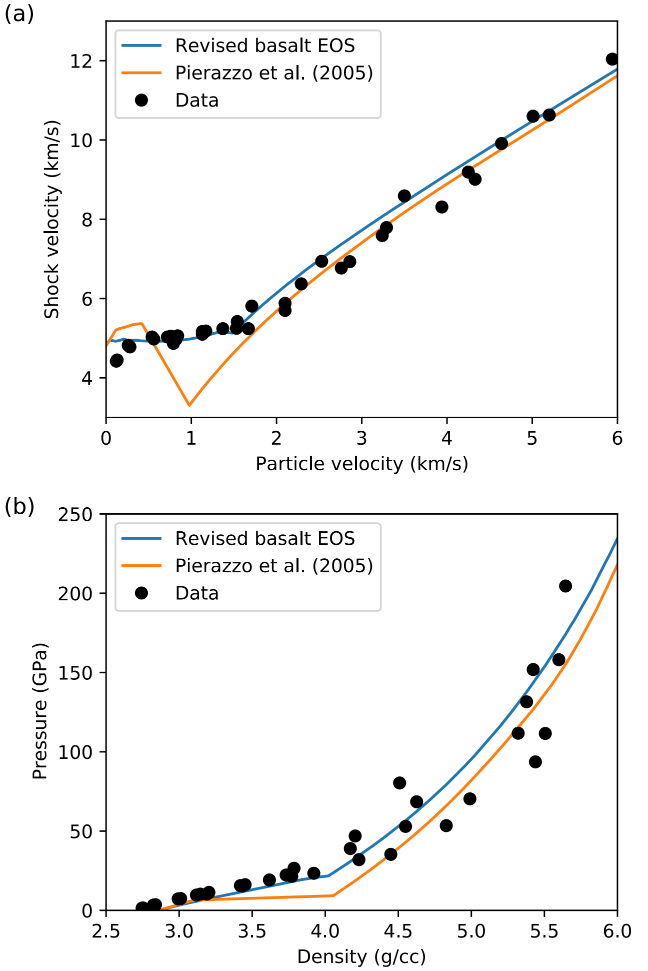


**Supplementary Figure 1. Comparison of old basalt EOS and revised basalt EOS**. Hugoniot curves in (a) shock velocity-particle velocity space and (b) pressure-density space for the revised basalt equation of state used in this work, compared with the previous ANEOS-derived basalt equation of state (Pierazzo et al., 2005) and experimentally derived Hugoniot data for basalt (van Thiel, 1977; Nakazawa et al., 1997).

The basalt EOS has a similar reference density to the grain density of lunar anorthosite and therefore should be a reasonable choice to simulate the behavior of the intact lunar crust[36,42,43]. Material strength parameters come from fits to gabbro rock strength[44] (Supplementary Table 1). It is important to note that the current implementation of dynamic fragmentation in iSALE only allows for one material type for each simulation. Each of the vertical impact runs was performed with a constant gravitational acceleration set to 1.62 $m/s^2$ to match that of the Moon and an impactor velocity of 15 km/s [35,39]. The input parameters are the same as they were in Ref. [19], and the minimum failure strain for material is also the same as Ref. [19]. Additionally, every

simulation, unless otherwise stated, was performed with a 14 K/km thermal gradient[49]. For more discussion of this please see the section below.

The resolution chosen for our 1 and 10 km diameter impactor runs was chosen to be 10 cells per projectile radius (cppr). This resolution allows us to finish our runs within a reasonable amount of time. For our largest impactor, 100 km in diameter, was run at 50 cells per projectile because we wanted to sufficiently resolve the upper crust. We also ran the 100 km diameter run with a resolution of 10 cppr and the results are similar, but with less variation and fine detail than the 50 cppr runs. This along with the testing of Ref. [19] give us confidence that a resolution of 10 cppr is sufficient. Additionally, we are not currently able to perform our simulations on multiple materials, due to limitations on the fragmentation model used. Therefore, a simulation using central gravity is not really possible, and we are not able to test the geometric effects of curvature for our simulations at this time.

**Supplementary Table 1. iSALE material input parameters**

| Parameter | Value | References |
|---|---|---|
| EOS | ANEOS Basalt | [36], see text |
| Poisson's Ratio | 0.25 | [47] |
| Melting temperature | 1513 K | [21,47] |
| Thermal softening coefficient | 1.2 | [21,47] |
| Simon A parameter | 1840 MPa | [47,48] |
| Simon C parameter | 7.27 | [47,48] |
| Frictional coefficient (damaged) | 0.71 | [21,47] |
| Frictional coefficient (undamaged) | 1.1 | [21,47] |
| Cohesive strength (damaged) | 0.01 MPa | [21,47] |
| Cohesive strength (undamaged) | 31.9 MPa | [21,47] |
| Strength limit (damaged) | 2.49 GPa | [21,47] |
| Strength limit (undamaged) | 2.49 GPa | [21,47] |
| k | $10^{36}$ | [19] |
| m | 9.5 | [19] |
| Thermal gradient | 14 K/km | [49] |

**Supplementary Table 2. Simulation parameters**

| Impactor Diameter (km) | Parameter | Value |
|---|---|---|
| 1 | cppr | 10 |
| | High-resolution area (cells) | X: 1 → 1200<br>Y: 201 → 1400 |
| 10 | cppr | 10 |
| | High-resolution area (cells) | X: 1 → 950<br>Y: 201 → 700 |
| 100 | cppr | 50 |
| | High-resolution area (cells) | X: 1 → 1000<br>Y: 401 → 1000 |

**Supplementary Text 2. Simulation with pre-impact porosity:**

While the preceding simulations were conducted on an initially nonporous lunar surface, the modern lunar crust contains significant porosity. To address modern lunar porosity evolution, we simulated a 1 km diameter impactor striking the lunar surface with an initial porosity of 6.8%, which is the approximate vertically averaged lunar crustal porosity[8] (Supplementary Figure 2). Pore space crushing at high pressure reduces porosity in material that lines the transient crater. This is consistent with previous work showing that pore space crushing dominates crater gravity signatures if pre-impact porosity is high[13]. However, the material located at greater depth further below and radial distance in the near surface regions experiences a significant increase in porosity (Supplementary Figure 2).

Our simulation including pre-impact porosity implies that successive impacts are able to generate porosity additively outside the crater rim. We expect that as the number of successive impact events increases so too does the deep lying tensile porosity. It is also important to note that the figures presented here do not represent the true final porosity field of the craters. Subsequent porosity modification occurring during crater collapse can significantly increase the porosity of the sub-crater material and rocks in the near-rim area[12,13]. However, the porosity generated outside the final crater rim should remain relatively unchanged from what is depicted here. The creation of porosity outside of large basins and crushing of porosity interior of smaller craters is consistent with independent work based on analysis of GRAIL gravity[14]. Accounting for evolution of porosity in this way during lunar bombardment would help us better understand the bombardment history of the moon[50]. Supplementary Figure 2 looks substantially different from Figure 1 in the main paper because the addition of pre-impact porosity considerably

changes the energy dissipation involved in the impact event compared to our other simulations into nonporous targets.

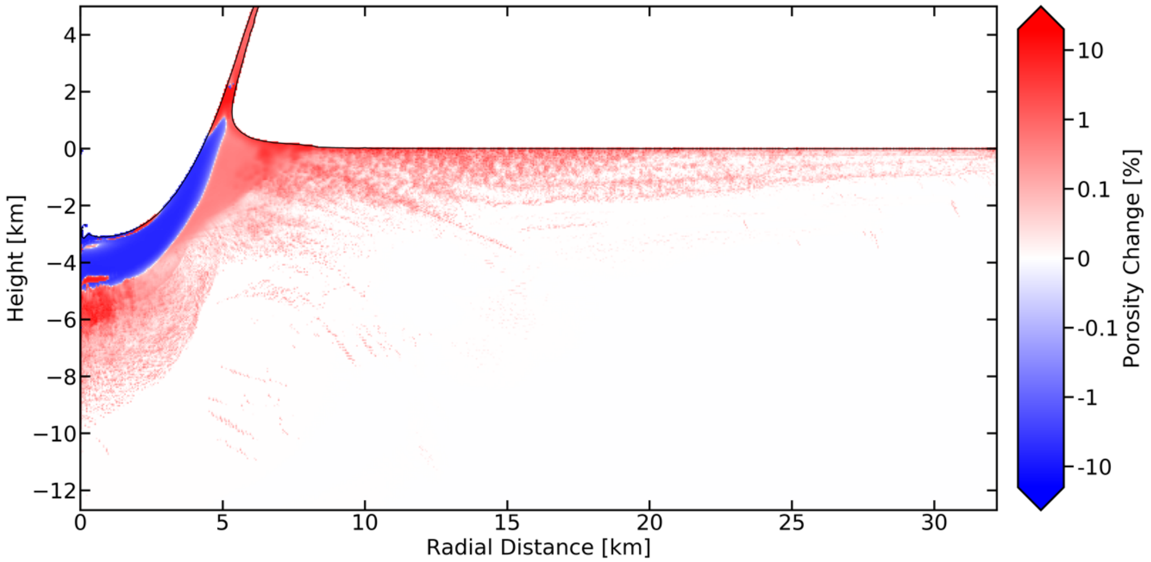


**Supplementary Figure 2. The resulting change in porosity after a 1 km diameter impactor struck a lunar surface with an initial porosity of 6.8%.** Material is colored according to change in porosity as indicated by the logarithmic color bar on the right. All material that is not colored (i.e., white) is material that has remained at the initial porosity of the crust, 6.8%. This result allows for direct comparison to results of previous work[13].

**Supplementary Text 3. Tensile Porosity and Thermal Gradient:**

To test the effects of thermal gradient on tensile porosity generation we used thermal gradients of 14 K/km for the Moon[49] and 30K/km, for our 100 km diameter impactor simulations into non-porous lunar-like surfaces at 15 km/s. The results of this are given in Supplemental Figure 3 with 14 K/km (Supplemental Figure 3a) and 30 K/km (Supplemental Figure 3b) showing negligible differences in their respective porosity structure. Though our results show little difference so shortly after impact, it may be important to note that viscous processes and crustal evolution will change these results significantly over time, with steeper thermal gradient bodies viscously relaxing more quickly.

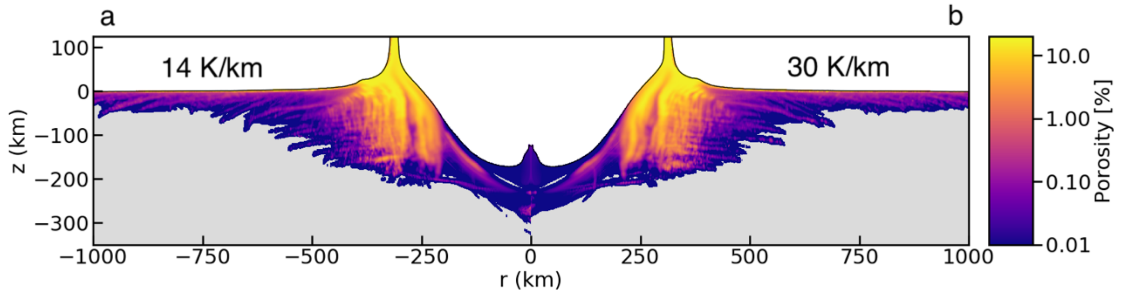


**Supplementary Figure 3. Mirrored plot of porosity after 100 km diameter impactors strike nonporous lunar-like targets with thermal profiles of 14 K/km (a, on left) and 30 K/km (a, on right).** Material is colored according to porosity percentage as indicated by the color bar on the right. All grey material is

nonporous. There is little difference between the porosity structures between the two different simulations.

**Supplementary Text 4. Further Comparison of New Tensile Routines to "Vanilla" iSALE-2D:**

To further illuminate the contribution the new tensile porosity and tensile fragmentation we have presented here Supplemental Figure 4, which shows the ratio of percent porosity gained in a simulation using the new routines over percent porosity gained in the same simulation with the latest stable release of iSALE-2D which does not utilize these new routines. In simpler terms, Supplemental Figure 4 represents the result of dividing Figure 1B by Figure 1A. It is important to note that this representation is not perfect and should be viewed qualitatively, as a direct comparison is not possible due to the fragmentation routine which randomizes defects into the mesh whereas the iSALE-2D Dellen does not. With that said, however, the largest difference in porosity gained, 10x – 100x, is located relatively deep within the originally nonporous crust, around 16-17 km below the surface.

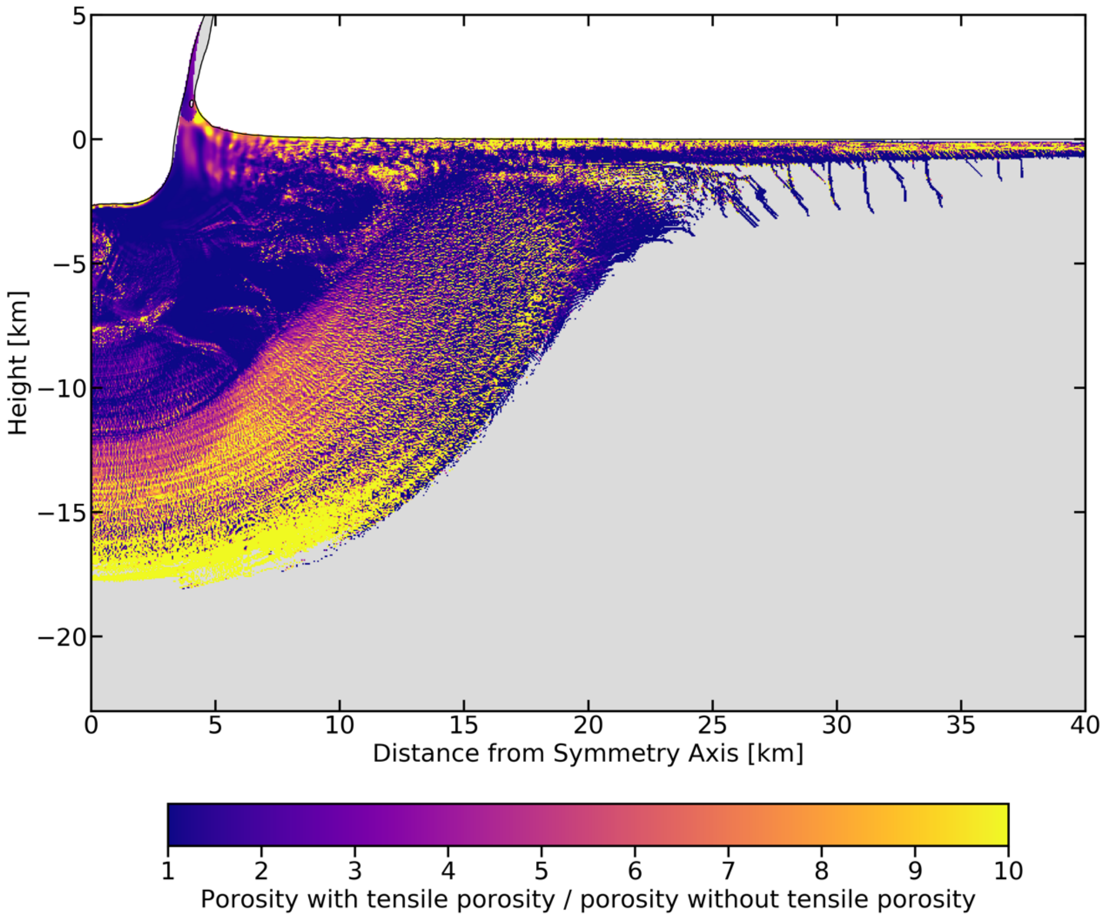


**Supplementary Figure 4. Further comparison of difference made by new tensile porosity routines.** Plot of the ratio of porosity generated with new tensile routines over porosity generated without the new tensile routines, for the otherwise same simulation of a 1 km diameter impactor striking a nonporous lunar-like target at 15 km/s. Material is colored according to the colorbar on the bottom, with all grey material representing nonporous material. It is important to note that this is not a perfect comparison as the two simulation results are inherently different, including spatially.

**References**

[42] Kiefer, W. S., Macke, R. J., Britt, D. T., Irving, A. J. & Consolmagno, G. J. The density and porosity of lunar rocks: the density and porosity of lunar rocks. *Geophysical Research Letters* **39**, (2012).

[43] Taylor, G. J. & Wieczorek, M. A. Lunar bulk chemical composition: a post-Gravity Recovery and Interior Laboratory reassessment. *Phil. Trans. R. Soc. A* **372**, 20130242 (2014).

[44] Thompson SL, Lauson HS, Melosh HJ, Collins GS and Stewart, ST, M-ANEOS: A Semi-Analytical Equation of State Code, Zenodo. doi: 10.5281/zenodo.3525030. (2019).

[45] M. van Thiel (Ed.), Compendium of shock wave data, *Lawrence Livermore Laboratory Report **UCRL-50108***, 719-729 (1977).

[46] S. Nakazawa, S. Watanabe, M. Kato, Y. Iijima, T. Kobayashi, and T. Sekine, Hugoniot equation of state of basalt. *Planet. Space. Sci.*, **v.45(11)**, p.1489-1492 (1997).

[47] Potter, R. W. K., Collins, G. S., Kiefer, W. S., McGovern, P. J. & Kring, D. A. Constraining the size of the South Pole-Aitken basin impact. *Icarus* **220**, 730–743 (2012).

[48] Wünnemann, K., Collins, G. S. & Osinski, G. R. Numerical modelling of impact melt production in porous rocks. *Earth and Planetary Science Letters* **269**, 530–539 (2008).

[49] Johnson, B. C. *et al.* Formation of the Orientale lunar multiring basin. *Science* **354**, 441–444 (2016).

[50] Huang, Y. H., Soderblom, J., Minton, D., Hirabayashi, M. & Melosh, J. Crustal porosity reveals the bombardment history of the Moon. (2021). *Under Review at Nature Portfolio.*
